# *Mycobacterium mucogenicum* Hand Infection in an Intravenous Drug Abuser

**DOI:** 10.1155/2018/1258649

**Published:** 2018-04-15

**Authors:** Sijan Basnet, Izza Mir, Rashmi Dhital, Garima Basnet, Nitin Patel

**Affiliations:** ^1^Department of Medicine, Reading Hospital, West Reading, PA 19611, USA; ^2^KIST Medical College, Lalitpur, Nepal

## Abstract

*Mycobacterium mucogenicum* is a rapidly growing mycobacterium found ubiquitously in water sources. It has been reported to cause widespread infections with infection entry from wound or central venous catheters especially in immunocompromised patients. Diagnosis is made from blood cultures which may take at least a week. Management includes removal of the source or drainage of wound infections and combination antimicrobial therapy.

## 1. Introduction

Nontuberculous mycobacteria (NTM) were first recognized as pathogens in the mid-1950s [1]. Rapidly growing mycobacteria (RGM) are a subset of NTM defined as mycobacteria that grow within 7 days [1, 2]. Many species of RGM are capable of causing disease in humans [1]. *Mycobacterium mucogenicum* belongs to the group of rapidly growing NTM, which are environmental organisms that survive in water distribution systems and tap water [3, 4]. The species was first identified in 1982 when 2 dialysis units described an outbreak of peritonitis with this agent under the name *M. chelonae*-like organism [5]. The current name, *M. mucogenicum*, is derived from the mucoid cell surface noted upon growth on the solid media. This mucoid surface provides the ability to form a protective biofilm, which facilitates infections of central venous catheters (CVC) and posttraumatic wounds, clinically the most common sites for *M. mucogenicum* infections [1, 2, 4]. The organism has also been isolated from respiratory secretions but has rarely produced clinical disease when found in this setting alone [1]. Moreover, these microorganisms have been shown to cause a wide spectrum of infections, including osteomyelitis, respiratory tract, bloodstream, and disseminated infections in both immunocompetent and immunocompromised hosts [6].

## 2. Case Presentation

A 46-year-old man with a history of heroin use presented to the emergency room with right hand pain, redness, and swelling four days after attempting to inject heroin into his vein but missing. The wound had progressively worsened to the point of severely limiting hand motion. The patient also reported fevers and chills. He was actively injecting heroin till the day of presentation but denied sharing needles. The past medical history was relevant for uncontrolled diabetes mellitus, hypertension, chronic hepatitis C infection, and chronic kidney disease stage III.

Upon presentation, the patient was afebrile (temperature: 100.3°F) with a pulse of 96 beats per minute (bpm), blood pressure of 177/96 mm Hg, respiratory rate of 13/min, and had 98% saturation on room air. Physical exam was unremarkable except for generalized erythema and swelling of the right hand with decreased gross sensation and power in the right hand.

**Figure 1:**
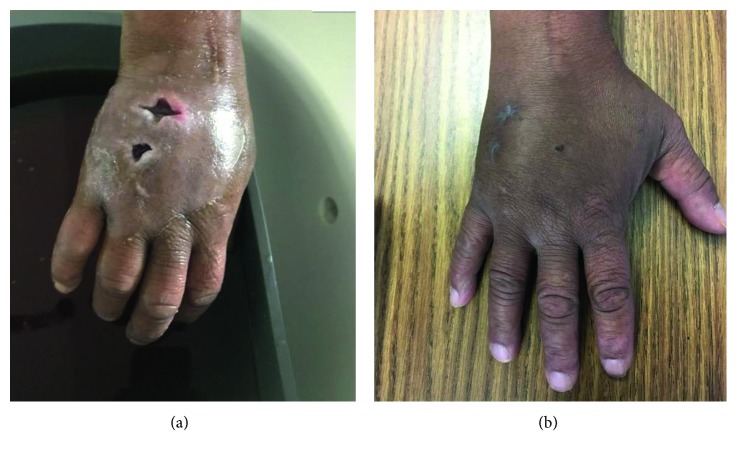
(a) Status postincision and drainage of abscess of the right hand. (b) Healed incisions of the right hand a month later.

White blood cell count was 10,800/*µ*L with 80.8% neutrophils. Lactate was 2.8 meq/l. Blood cultures were sent, and the patient was started on intravenous (IV) vancomycin, piperacillin-tazobactam, and fluids for sepsis management. X-ray of the right hand showed dorsal soft tissue swelling without any other acute abnormalities or radiopaque foreign body. Urine drug screen was positive for heroin, opiates, and benzodiazepines. HIV screen was negative. Plastic surgery performed 2 incisions on the dorsum of the right hand which drained clear fluid without pus. Counterincision was made on the distal part on the dorsal aspect of the hand (Figure 1(a)). Cultures were taken from the deep wounds, and the wounds were packed. The patient left against medical advice but was called back after a day when 1 of his 2 blood cultures was positive for methicillin-susceptible *Staphylococcus aureus* (MSSA). His wound culture was also positive for MSSA. Upon return, he was afebrile (temperature: 97.9°F) with a pulse of 100 bpm, blood pressure of 177/96 mm Hg, respiratory rate of 13/min, and had 99% saturation on room air. He had persistent pain but was able to move his fingers. He acknowledged inhaling heroin before coming in. He was started on intravenous ampicillin/sulbactam, and blood cultures were sent on day 1 of second admission. White count was 11,400/*µ*L with 70.5% neutrophils. Transthoracic echocardiogram was negative for vegetation.

Seven days after initial presentation to the hospital, blood cultures from the first day were reported to be positive for acid fast bacilli (AFB). Repeat cultures from day 1 of second admission also came back positive for AFB. TB QuantiFERON (negative), CD4 count (610/*µ*L), chest X-ray (negative for consolidation), and HIV viral load (negative) were done. On day 10, *M. phocaicum* was identified preliminarily via best match (CPAL: Central Pennsylvania Alliance Laboratory). Unfortunately, no wound cultures were sent for AFB testing. IV amikacin and cefoxitin with probenecid were started, and intravenous ampicillin/sulbactam was discontinued. Questioning to determine a source of infection revealed that the patient had used tap water and phenol as a solvent for heroin. Repeat blood cultures were negative. *Mycobacterium* was later confirmed as *M. mucogenicum.* Amikacin was stopped due to acute kidney injury, and the patient was instead put on meropenem and cefoxitin for 2 weeks followed by doxycycline 100 mg twice daily and trimethoprim-sulfamethoxazole (TMP-SMX) daily for 6 weeks. On follow-up, he had completed full course of antibiotics and his hand had improved significantly (Figure 1(b)).

## 3. Discussion

*M. mucogenicum* is ubiquitously found in public and hospital tap water, as well as in shower faucets and environmental surfaces. There have been several case reports linking *M. mucogenicum* infections to potable water sources [1, 7, 8]. The most significant diseases caused by *M. mucogenicum* are central venous catheter and wound infections in immunocompromised persons [1, 8]. Equipment exposed to contaminated water can lead to nosocomial bacteremias, including central venous catheter-related and hemodialysis-related infections, as the bacteria are resistant to many commonly used hospital disinfectants [1, 2]. Kline et al. described an outbreak of *M. mucogenicum* bacteremia in bone marrow transplant recipients and oncology patients caused by water contamination of central venous catheters during bathing [8]. Case reports have also described *M. mucogenicum* infections of the central nervous system in immunocompetent hosts, skin lesions in the presence of the tumor necrosis factor-*α* antagonist etanercept, peritonitis associated with peritoneal dialysis, bacteremia in patients with hepatitis B and D associated cirrhosis with negative ascites fluid cultures, bone marrow involvement with retinal lesions and acute respiratory distress in a patient with idiopathic CD4+ T lymphocytopenia, and wound infections [2]. *M. mucogenicum* is the most common RGM implicated in central line-associated bloodstream infections [1]. Undertreatment of contaminated water sources has been implicated in infections including suboptimal chlorination of water and hemodialyzers treated with formaldehyde concentrations below those required to prevent growth of *M. mucogenicum* [4, 9].

Due to slow growth of mycobacteria, diagnosis is often difficult and delayed. Appropriate blood cultures are critical and may need verification with repeat cultures [9]. Differentiation of mycobacteria to the species level is also complicated due to the slow growth rate of mycobacteria. Phenotypic and biochemical test results may vary depending on the growth conditions, sometimes leading to inaccurate results [7]. More importantly, as *M. mucogenicum* is an organism that resides in water bodies and soil, a complete environmental assessment needs to be performed. It is crucial to collect water samples from dialysis machine, RO, and incoming water supply [9].

## 4. Treatment

RGM are difficult to eradicate from venous catheters since they survive by biofilm formation. Successful therapy requires removal of the catheter or other infected devices in addition to appropriate antimicrobial agents [1, 4, 9]. Management of *M. mucogenicum* infections includes both removal of indwelling catheters, similar to management of other RGM, and combination antimicrobial therapy [2]. RGM are usually resistant to standard antimicrobial therapy, but *M. mucogenicum* is generally more susceptible to antimicrobials [1]. However, like other RGM, it is resistant to first-line antituberculosis agents [1]. El Helou et al. found *M. mucogenicum* most susceptible to antibiotics including 100% susceptibility with amikacin, clarithromycin, imipenem, and TMP-SMZ [6]. Acquired resistance of RGM to certain agents such as ciprofloxacin has been reported, necessitating use of multidrug therapy in most cases [1]. El Helou et al. found that use of combination antimicrobial regimens was superior to monotherapy and tended to be associated with lower relapse rate [6]. The current NTM practice guidelines do not explicitly state a specific treatment protocol for *M. mucogenicum* but do state that most isolates are susceptible to multiple antimicrobial agents including aminoglycosides, cefoxitin, clarithromycin, minocycline, doxycycline, quinolones, TMP-SMX, and imipenem [1, 2, 4, 9]. Although the duration of treatment of nondisseminated RGM infection has not been established in the literature, prolonged therapy for several weeks to months appears to be necessary to eradicate infection [1]. Gaviria et al. found that patients with catheter exit site infection were successfully treated with catheter removal and an average of 2 antibiotics administered for 2–4 weeks [10]. Most antimicrobial regimens for catheter tunnel infection or catheter-related bacteremia consisted of an initial phase with 2 antimicrobials administered intravenously(e.g., amikacin, imipenem/cilastatin, cefoxitin, and tobramycin) for 2–4 weeks, followed by 2 antimicrobials administered orally (e.g., clarithromycin, ciprofloxacin, and doxycycline) for 4–6 weeks [10]. Severity of infection and underlyingimmune compromising condition and/or degree of immune suppression need to be factored in when implementing longer duration of combination antimicrobials [4]. In addition, the guidelines also mention that care should be taken when using macrolides in combination with immunosuppressant therapy owing to drug-drug interactions [2]. Empiric treatment must be initiated with multiple agents while pursuing culture and sensitivity testing [1]. The decision to use combination antimicrobial therapy should be dictated by susceptibility testing, the patient's tolerance of the drugs, and the specific clinical scenario [2].

## 5. Conclusion

RGM are emerging pathogens in the immune-compromised host [4]. *M. mucogenicum*, a rapidly growing NTM present in contaminated water, should be recognized as a potential source of infection, especially in the immunocompromised population [2]. *M. mucogenicum* infection should be considered early in the care of transplant patients or of otherwise immunocompromised patients with suspected catheter-related bloodstream infection or peritonitis.
